# High-Performance Material for the Effective Removal of Uranyl Ion from Solution: Computationally Supported Experimental Studies

**DOI:** 10.1021/acs.langmuir.2c00978

**Published:** 2022-08-10

**Authors:** Selçuk Şimşek, Yavuz Derin, Savaş Kaya, Zeynep Mine Şenol, Konstantin P. Katin, Ali Özer, Ahmet Tutar

**Affiliations:** † Faculty of Science, Department of Chemistry, Sivas Cumhuriyet University, 58140 Sivas, Turkey; ‡ Department of Chemistry, Sakarya University, 54050 Sakarya, Turkey; § Health Services Vocational School, Department of Pharmacy, Sivas Cumhuriyet University, 58140 Sivas, Turkey; ∥ Zara Vocational School, Department of Food Technology, Sivas Cumhuriyet University, 58140 Sivas, Turkey; ⊥ Institute of Nanoengineering in Electronics, Spintronics and Photonics, National Research Nuclear University “MEPhI”, Kashirskoe Shosse 31, Moscow 115409, Russia; # Engineering Faculty, Metallurgical and Materials Engineering Department, Sivas Cumhuriyet University, 58140 Sivas, Turkey

## Abstract

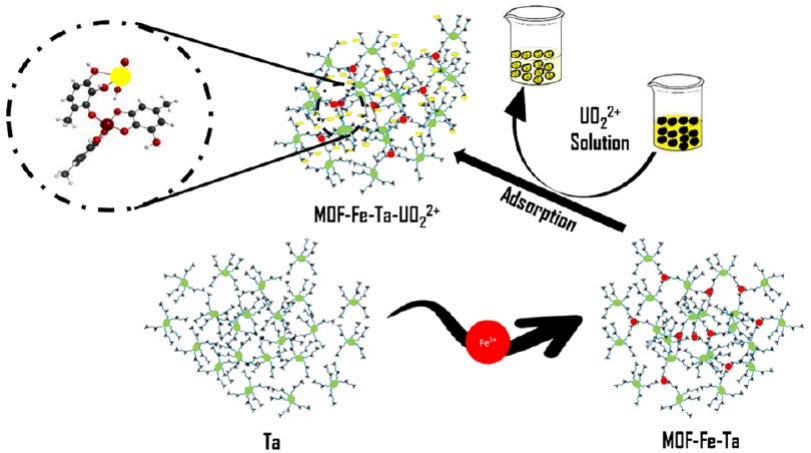

Adsorption is a widely used method for pollution removal and for the recovery of valuable species. In recent years, the use of metal–organic compounds among the adsorbents used in adsorption studies has increased. In this study, the performance of the water-insoluble Fe complex as a metal organic framework (MOF-Fe-Ta) of water-soluble tannic acid, which is not used as an adsorbent in uranium recovery and removal, was investigated. For the characterization of the new synthesized material, Fourier transform infrared, scanning electron microscopy, and X-ray diffraction analyses were performed. The changes in the adsorption process based on various parameters were investigated and discussed. The point of zero charges value of the adsorbent was found as 5.52. It was noticed that the adsorption increases as the pH increases. Analyzing the effect of concentration on adsorption, we determined which model explained the adsorption better. The monolayer capacity of the adsorbent determined in light of the Langmuir model was reported as 0.347 mol kg^–1^. The Freundlich constant, namely the β value obtained in the Freundlich model, which is a measure of surface heterogeneity, was found to be 0.434, and the *E*_DR_ value, which was found from the Dubinin–Raduskevich model and accepted as a measure of adsorption energy, was 10.3 kJ mol^–1^. The adsorption was kinetically explained by the pseudo-second-order model and the adsorption rate constant was reported as 0.15 mol^–1^ kg min^–1^. The effect of temperature on adsorption was studied; it was emphasized that adsorption was energy consuming, that is, endothermic and Δ*H* was found as 7.56 kJ mol^–1^. The entropy of adsorption was positive as 69.3 J mol^–1^ K^–1^. As expected, the Gibbs energy of adsorption was negative (−13.1 kJ mol^–1^ at 25 °C), so adsorption was considered as a spontaneous process. Additionally, the power and mechanism of the interaction between studied adsorbent and adsorbate are explained through density functional theory computations. Computationally obtained data supported the experimental studies.

## Introduction

The rapid rise of industrialization and population growth in the last century has led to an increase in the demand for energy and raw materials use. This situation has also brought about an increase in pollution in the environment. The inadequacy of fossil fuels has led countries to seek alternative energy sources. Nuclear energy is among the ones with a high preference rate among alternative energy sources. The uranium element, which is among the raw materials of nuclear energy, is among the precious species in this respect. However, uranium enters the waters for many reasons such as nuclear facilities, scientific research laboratories, widespread use of uranium in many industries, thermal power plants as well as natural causes such as volcanic eruptions and has a negative effect on the environment and to human health. Therefore, it is especially important to remove uranium from wastewater. Considering its concentration in seawater, its recovery as a raw material is also economically important.

Nowadays, many physicochemical or biological methods to remove metallic and organic wastes as well as recovery of valuable species from aqueous environments are used.^1−4^ However, for a method to be widely used, it must be sustainable, economical, practical, and reproducible. When evaluated from this point of view, recovery–removal by adsorption stands out among methods such as precipitation, membrane filtration, and biological increment.^5^ The design of the adsorptive materials is important in the removal of pollutants by adsorption. Properties such as adsorption capacity, adsorption speed, selectivity to the species to be adsorbed, and economic and practical use are decisive in the selection of an adsorbent. In addition to the commonly used adsorbents like carbon, clays, zeolite, and natural and synthetic polymers, other materials with improved properties, such as composite metal organic frameworks (MOFs), have found wide application in the adsorption field.^6−8^

MOFs are multifunctional materials having metallic centers and organic ligands bonded via robust coordination bonds.^9^ They numerous applications like sensing of chemicals, catalysts, drug delivery, gas storage and separation, etc.^10,11^ Moreover, these materials have gained attention as adsorbents for the removal of organic and inorganic species because of their porous structure and functional groups on organic units. By selection of ligands and metals, MOFs can be designed with different pore sizes and surface functionalities, rendering them to adsorb numerous ions or molecules.^12−14^

**Figure 1:**
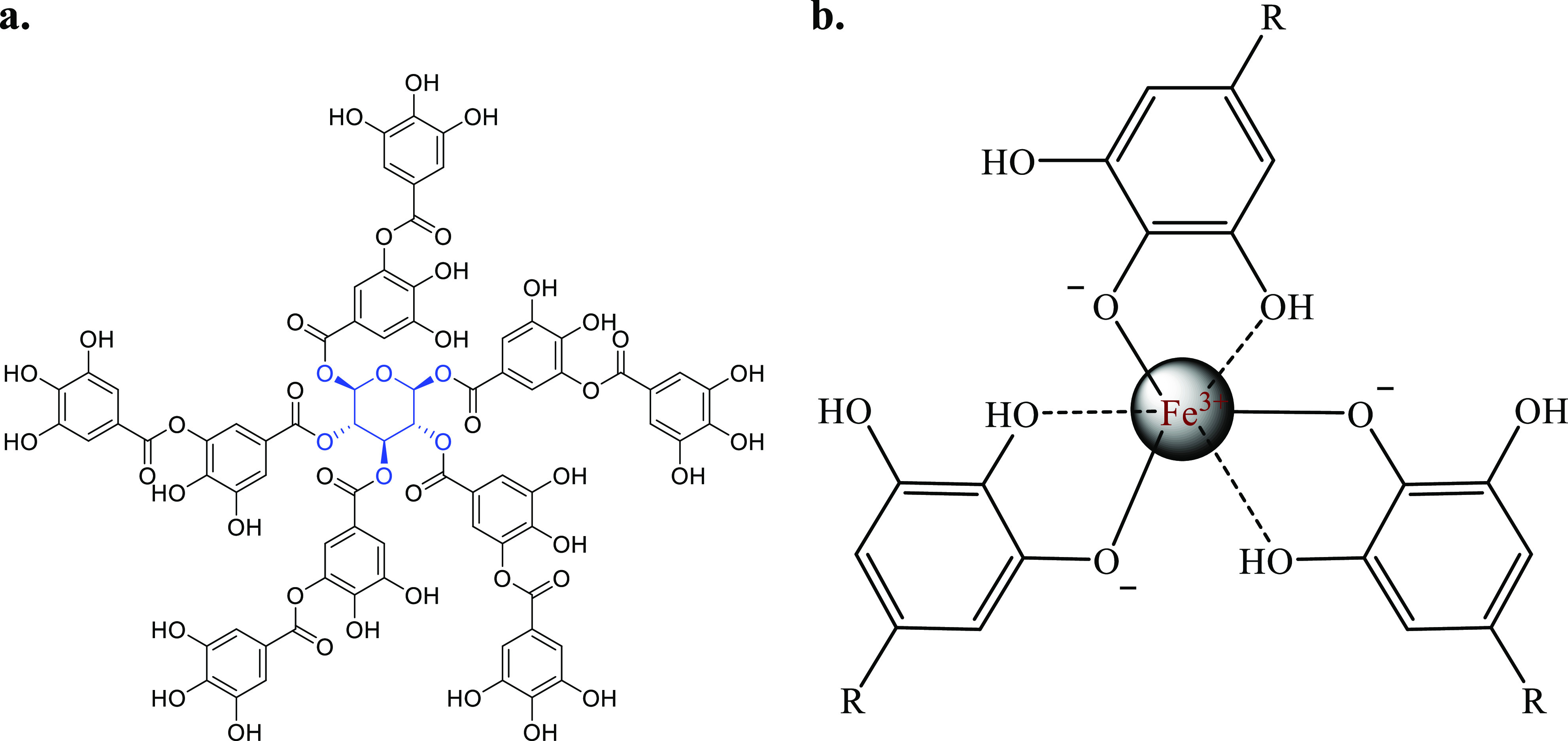
(a) Structure of tannic acid. (b) Iron-tannic acid complex.

Tannic acid (Figure 1a) is among the most abundant and well-known polyphenolic molecular structures in nature that consists of glucose at the center linked to gallolyl residues via ester bonds. Tannins are abundant in plants such as grapes and bananas as well as beverages like black tea, green tea, wine, and beer.^15^ These compounds are known as macromolecules having a large number of hydroxyl groups. The structures with hydroxyl and carboxyl groups can easly form chelates with metal ions. However, their high water solubility in a broad pH range prevents use as an adsorbent, but it has been reported that metal tannates such as zinc, titanium, zirconium, and iron are almost insoluble in water, so they can be used as adsorbents for ions and molecules in water environments. Due to these properties, tannic acid based MOFs are promising materials for an adsorbent that is economic, eco-friendly, and easy to produce.^16−19^

In this report, the complex of tannic acid formed with Fe (Figure 1b) was synthesized and its characterization clarified by scanning electron microscopy (SEM), Fourier transform infrared (FTIR), and X-ray diffraction (XRD) analyses. The surface charge characteristic was elucidated using the point of zero charges (PZC) method. The adsorption property of the newly synthesized material was tested for the uranium ion, and the effects of the factors closely related to the adsorption like concentration, time, pH, and temperature were analyzed and reported as comprehensive within the scope of the study. We investigated the change of adsorption in different pH values, temperatures, and uranyl ion concentrations. Additionally, we analyzed how adsorption changes with the time. Based on density functional theory (DFT) computations, the adsorption mechanism and the nature of the interactions were highlighted. Theoretically obtained results and experimental insights show that the newly synthesized material has a very high affinity for uranium.

## Materials and Methods

### Chemical Substances and Devices

In this study, FeCl_3_·6H_2_O, NaHCO_3_ HCl, NaOH, KNO_3_, and (CH_3_COO)_2_UO_2_·2H_2_O were bought from Merck (Germany). Tannic acid and the other chemicals used were purchased from Sigma-Aldrich.

The concentration of uranyl ions was analyzed via a Shimadzu UV–vis spectrophotometer. It is well-known that this spectrometer with a wavelength accuracy of ±0.2 and 2 nm over a wide wavelength range is widely preferred in such an experimental process. To check the important functional groups in the structure, the FTIR analysis was carried out with a PerkinElmer Spectrum two ATR FT-IR. While pH measurements were made with a Selecta pH meter, a Hettich centrifuge device was used for the centrifugation processes. To work at a constant temperature, a Nuve NT 120 thermostat was used.

SEM analysis was conducted with a TESCAN Mira3 XMU FEG (Brno, Czechia) with a 10 kV accelerating voltage and a 10 mm working distance. The powders were poured on a double-sided carbon tape on an aluminum stub, and the residue was cleaned by an air gun. To produce a conductive surface, 5 nm of gold was coated by a Quorum Q150R ES magnetron sputter (Birmingham, UK). SEM analysis was performed with a backscattered detector (BSE) to evaluate possible different phases or atoms to be adsorbed on MOF-Fe-Ta. Energy dispersive spectroscopy (EDX, Inca x-act, Oxford Inst, UK) was conducted on a flat surface of powders with a 10 mm analytical distance and 10 kV as in SEM investigations to identify U related species semiquantitatively. XRD analysis was performed with a Rigaku X-ray diffractometer (RSGD T01453).

### Preparation of Fe-Tannate Complex

The iron-tannate complex was prepared as described in the literature.^18^ Briefly, 10 mL of an aqueous solution of commercially available tannic acid (0.1 M) was drop-wise added to a well-stirred solution of 20 mL of FeCl_3_·6H_2_O (1M) under continuous stirring. Later, using a sodium bicarbonate solution, the studied pH was fixed to 7. The final mixture was mixed for 2 h. Then the resultant was centrifuged at 3000 rpm for 3 min. Washing and drying processes of the complex were carried out, respectively.

### Adsorption Experiments

In all adsorption experiments, we used the batch method. Solutions to be used in the adsorption experiments were prepared by adding 10 mL of a 400 mg L^–1^ UO_2_^2+^ ion solution to a solution of 50 mg of MOF-Fe-Ta in 10 mL of a polypropylene solution at pH 4.5. These were allowed to reach equilibrium at 140 rpm for 24 h, after which the aliquots were withdrawn and filtered. To analyze the concentration of UO_2_^2+^ ions, the PAR method^20^ was considered. In this method, a complex with PAR (4-(2-pyridylazo) resorcinol) at pH 8.5 of uranyl ions was obtained. A 3.5 × 10^–3^ M PAR solution was prepared using a buffer solution [Tris/HCl buffer solution with pH 8.5, 0.7 M]. To compute the adsorption % *Q* (mol kg^–1^) and % desorption, the following formulas were used:
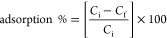
1
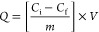
2
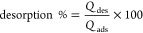
3where *C*_i_, *C*_f_, and *m* are first and final concentrations of the UO_2_^2+^ ion and the MOF-Fe-Ta mass (g), respectively. *V* represents the volume value of the solution.

## Results and Discussions

### FTIR Analysis

**Figure 2:**
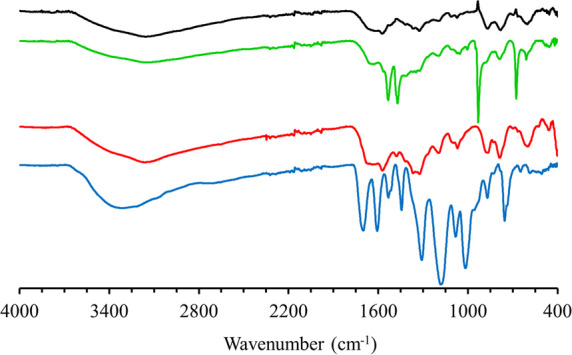
FTIR spectra of the (a) tannic acid (blue), (b) MOF-Fe-Ta complex (red), (c) uranyl adsorbed MOF-Fe-Ta (green), and (c) MOF-Fe-Ta after regeneration (black).

FTIR spectroscopy was performed for the structural characterization of the adsorbent (Fe-tannate) compared with starting material, pure tannic acid. Moreover, the interactions between the adsorbent and uranyl ions were demonstrated. After recovery of the uranyl ions, it was confirmed that the adsorbent’s FTIR spectra matched with the one before adsorption. FTIR spectra of tannic acid, Fe-tannate before and after uranyl adsorption, and uranyl adsorbed Fe-tannate is illustrated in Figure 2. The broad peak between 3500 and 3000 cm^–1^ was observed, which is an indication of phenolic OH groups and aromatic C–H stretching should have seen at this range, but this can be overlapped with OH stretching. The peak noticed at 1320 cm^–1^ is because of phenolic hydroxyl groups. The peaks at 1530 and 1440 cm^–1^ are due to the C=C stretching of tannic acid. Phenolic C—O stretching appeared at 1160 cm^–1^.^21,22^ After complexation of tannic acid with iron, the peaks were slightly shifted and their intensities decreased. A peak appeared at 597 cm^–1^ due to Fe—O bond formation.^23,24^ After UO_2_^2+^ adsorbed on new designed adsorbent system, four new and pronounced peaks were observed. The peaks at 1533 and 1470 cm^–1^ can be related to carboxylate groups in the structure. Additionally, the peak at 930 cm^–1^ originated from the stretching vibration of linear structure of uranyl ions ([O=U^IV^=O]^2+^). The peak located at 677 cm^–1^ can be the complexation of phenolic oxygen with uranyl.^25,26^

### SEM Analysis

**Figure 3:**
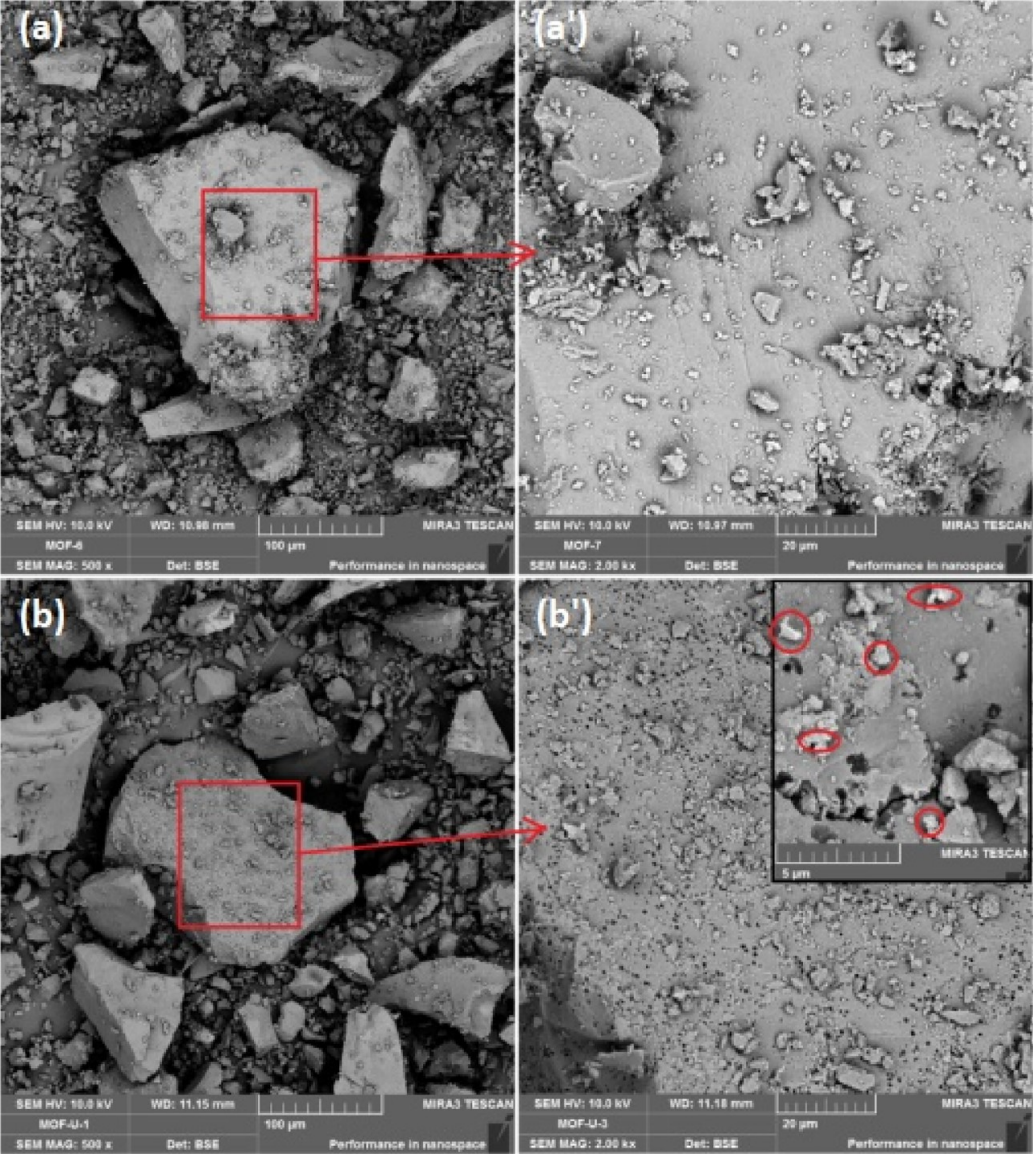
SEM images: (a)/(a′), the pure MOF-Fe-Ta; (b)/(b′), UO_2_^2+^ adsorbed MOF-Fe-Ta.

It can be understood from Figure 3(a) and (a′) the MOF-Fe-Ta samples exhibit different particle distributions with irregular shapes in the range of 10–200 μm. The MOF-Fe-Ta surface is a brittle type fractured morphology as being an organic material Fe centered. MOF-Fe-Ta becomes solution sensitive, which may be attributed to be solved by an acid or basic environment by having Fe in the centers. Since it is bare MOF-Fe-Ta, due to the carbon tape underneath, it can be seen as more white. However, in (a′), there is no phase difference on the surface due to no extra phase. For (b′), it is evident to have a phase contrast on the surface due to the presence of denser phases as U–O related species where they show darker and brighter regions.

From Figure 3(b) and (b′), the dimensional stability of MOF-Fe-Ta by U doping changes and the surfaces of the powders become brighter. Most particles are about 200 μm in size, while there are also many particles about 70 μm in size. Additionally, particles smaller than 10 μm are still present, albeit in very small quantities. The surface of bare MOF is seen as very smooth due to polymerization and brittle features. As it was doped by U–O species, it is noteworthy the intense pore structure is evident. As Fe is in the center of MOF-Fe-Ta, there occurred some holes like pores on the surface of <1 μm in diameter, which may be attributed to Fe loss. Because the red circles are found to be U species, the substitution of Fe with U from the solution may occur. These holes may also favor the fracture of bigger particles to produce average size particles of ∼70 μm due to the crack intensity increase of the inorganic structure as ceramics around pores while precipitating from solution.

**Figure 4:**
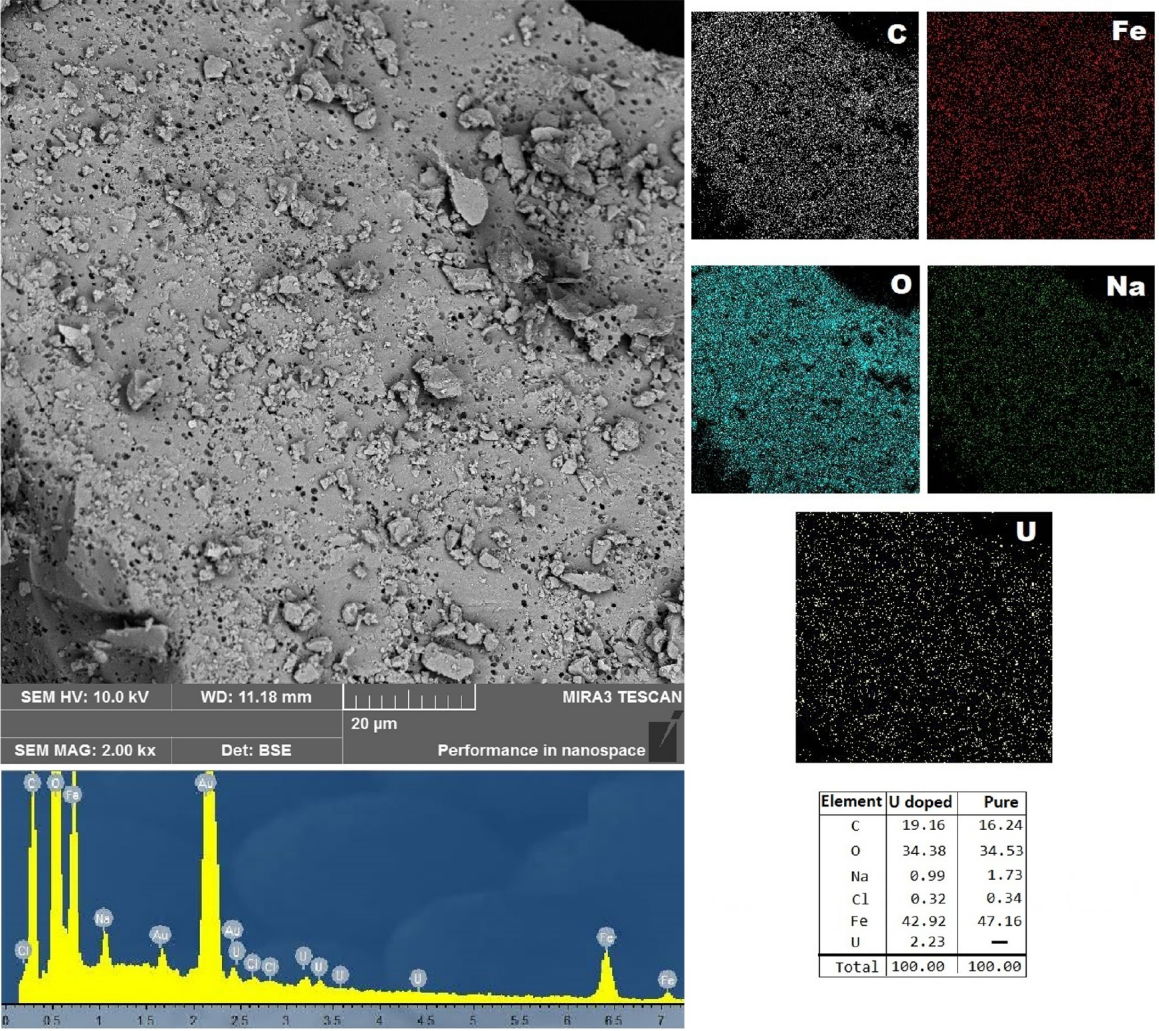
SEM-EDX analysis of pure and UO_2_^2+^ adsorbed MOF-Fe-Ta spectra.

From the EDX analysis in Figure 4, MOF-Fe-Ta was proved to have Fe centers along with C and O. MOF-Fe-Ta was doped with U species as seen from the map spectrum and quantification. The U doping is seen as well distributed and has 2.23 wt % on the MOF-Fe-Ta surface. Fe may remain on the surface pores by U doping and react with the liquid medium prior to U adsorption, which in turn may result in Fe loss on the surface seen by holes. Possibly, U takes the place of Fe in the MOF-Fe-Ta surface. Since the surface has pores smaller than 1 μm, this may be concluded as the loss of Fe while U penetrates to the vacancy of Fe.

### XRD Analysis

**Figure 5:**
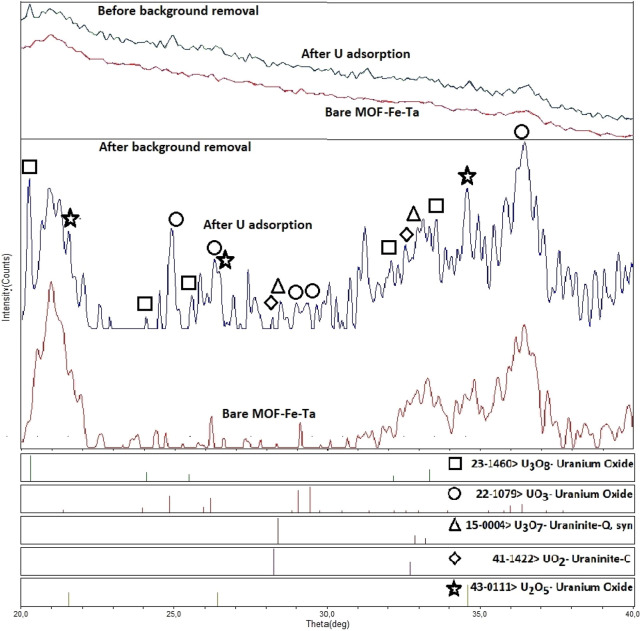
XRD pattern of MOF-Fe-Ta and UO_2_^2+^ adsorbed MOF-Fe-Ta samples.

Figure 5 shows the XRD patterns of MOF-Fe-Ta prior to background removal and after background removal of patterns for bare MOF-Fe-Ta and subsequent U doping. As seen in the pattern without background removal, MOF-Fe-Ta is most likely a hump-like organic structure pattern due to its low crystallinity while having some crystallization peaks originate from the volumetric crystallization of C–H–N–O species via the centered Fe.^27^ It is evident that a bare MOF has a suppressed peak series without U doping, where U species make the polymeric structure more crystallized locally to produce U–O related phases. One may not be able to see the all phases in detail unless decreasing the background. After doping with U, a significant difference occurred due to U–O peaks that originated from the adsorption of U on MOF-Fe-Ta. To evaluate the differences better, the background of the peaks was taken by a linear fit, and below peak list was seen as “After background removal”. It is clearly seen as a rectangle sign the peaks at 20.3°, 24.2°, 25.5°, 32.3° and 33.2° 2θ were directing the U_3_O_8_ with a JCPDS file of 23-1460. This uranium-oxide is a multivalent combined structure of U_2_O_5_ and UO_3_ and have +5 and +6 valences of U, respectively.^28^ This is well understood to be widely found in the surface by being more in volume by higher peak intensities and sharper peaks. The precipitation of this phase is favorable from solutions by acidic environments due to different rates of oxidized species to precipitate might statistically combine them together. The peaks at 24.9°, 26.1°, 29.2°, 29.4° and a split peak at 36.2° belong to UO_3_ as shown by circles with a JCPDS file #22-1079. This excess oxygen compound (UO_3_) would be produced by gaining oxygen to U_3_O_8_ in any part of the precipitation or adsorption process.^29,30^ The possible oxidation reaction could be as follows
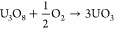
R1Uraninite-Q as U_3_O_7_, shown by triangles, with a JCPDS file #15-0004, could have been considered another oxygen deficient phase. Uraninite-C was shown by diamonds with JCPDS file #41-1422, to be consumed during the adsorption process that has the lowest volume amount among the phases. UO_2_ was the main ion to be used in experiments, which cannot stand still while oxidizing or deoxidizing reactions happened.^31^ The U_3_O_7_ phase could be evaluated as oxygen deficient from the U_3_O_8_ viewpoint, while it is an oxygen gaining phase from the UO_2_ viewpoint, which can be concluded as reactions as follows
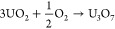
R2or
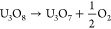
R3The U_2_O_5_ phase, shown by stars, with a JCPDS file #43-0111, is one of the most common compounds, seen after adsorption, and also can be concluded as oxygen gaining from the UO_2_ side while it is oxygen deficient from the UO_3_ side as shown by possible reactions as follows
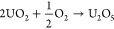
R4or
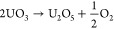
R5

The main peaks for nano crystallites are not of interest. It can be reported that very low nano sized (<10 nm) oxides cannot be related to the main peak’s direction of most preferred orientations of a polycrystalline powder. The nano grains are formed on the surface by adsorption on the surface, and the orientation of precipitation could be other than the main peak as seen from SEM-EDX elemental mapping, which was well distributed along the surface about a few nanometers in diameter. This would also prove the presence of U–O related phases especially adsorbed onto the surface that can affect XRD. As described in the equation formulations presented, the combined phases of two main compounds either by gaining or losing oxygen, such as U_3_O_8_, UO_3_, and U_2_O_5_, are the main compounds of the surface in Figure 5. The U–O related species in high intensities originated from from the increasing of U concentration on surface of MOF-Fe-Ta.

### Effect of pH and PZC for MOF-Fe-Ta

**Figure 6:**
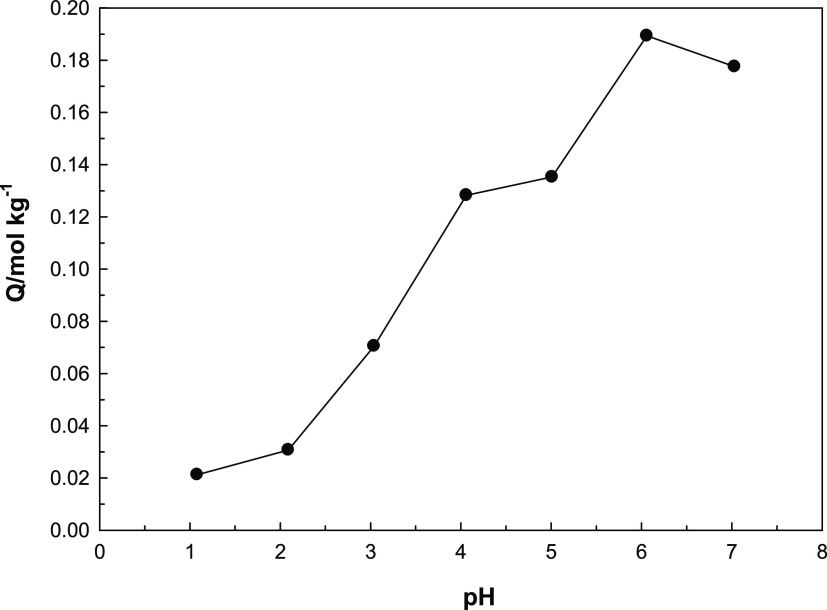
Graph showing pH effect on adsorption of UO_2_^2+^ onto MOF-Fe-Ta.

In such studies, the pH of the studied solution is one of the remarkable parameters. The solution pH is important for two reasons. First, it can change the interaction with the surface by affecting the types of ions or molecules in the solution. For example, polyanionic species formed with increasing pH in metal ion adsorption cause both precipitation of ions and a decrease in the adsorption. The second effect is on the surface. The proportional increase of H^+^ or OH^–^ ions in the solution medium can make the surface positive or negative. Although most of the adsorption studies are performed at the natural pH of the studied systems, the optimum pH research gains importance in adsorbent regeneration or adsorbate recovery studies. Optimum pH research was conducted, and Figure 6 shows the results obtained. It is clear from the figure that adsorption of uranyl on the new adsorbent system increases as pH increases. This result can be attributed to the decrease in the cationic groups on the surface together with the numerical decrease of the H^+^ ions in the environment with increasing pH, and accordingly the decrease in the repulsion forces between the adsorbed cationic uranyl ions and the surface. In addition, with an increasing pH, the substitution of polycationic species instead of the dominant UO_2_^2+^ cation at a low pH leads to increased adsorption.^32^ Adsorption studies could not be performed in alkaline conditions because the precipitation of polyanionic species and hydroxides formed under these conditions adversely affects adsorption.^33^ Since the natural pH of uranyl is in the range of 4–5, it has been observed that this adsorption pH can be also used for this new material.

**Figure 7:**
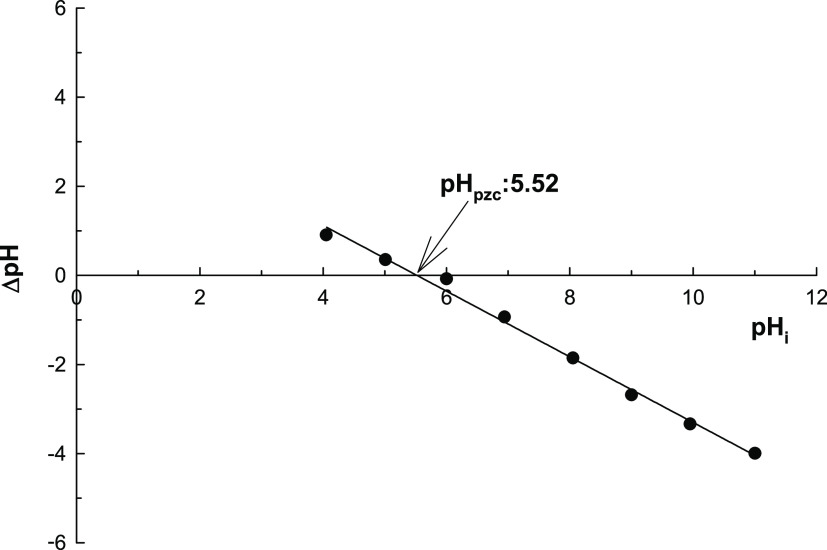
PZC for MOF-Fe-Ta.

It is well-known that PZC is reported as the solution pH value that the adsorbent has zero surface charge.^34^ To find the PZC value of the new designed adsorbent system, the new material was kept in the solution having 0.1 mol L^–1^ KNO_3_ in the pH = 1.0–12.0 range during a 24 h period, and then equilibrium pH values for all solutions were noted. pH adjustment was made with the help of 0.1 M HCl or NaOH solutions. The PZC value can be found via the linear relation between pH_i_ and ΔpH. Here ΔpH represents the difference between the initial pH_i_ and final pH_f_. It is clear from Figure 7 that surface charge of the new designed material MOF-Fe-Ta was determined as 5.23.

### Effect of Adsorbent Dosage

**Figure 8:**
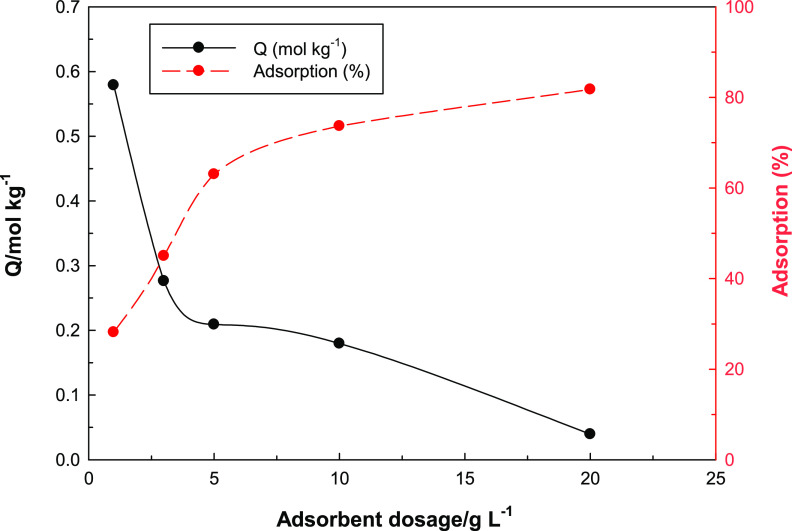
Graph showing the effect of adsorbent dosage on adsorption of UO_2_^2+^ onto MOF-Fe-Ta.

One of the commonly used parameters in adsorption research is the determination of the amount of adsorbent used. Naturally, the adsorption will increase with the amount of the adsorbent. However, especially in chemical adsorption, when the adsorption centers and the liquid–solid interface are evaluated together, the adsorption reaches saturation above a certain amount. Above this amount, the amount of adsorbed species is independent of the amount of the adsorbent. The variation of the composite uranyl ion adsorption study with the adsorbent mass was studied, and the result is shown in Figure 8. As can be seen, the adsorption increases with the increasing amount of the adsorbent, but then it reaches a plateau. The amount of the adsorbent at the point where it reaches the plateau was selected, and other parameters were studied at this amount of the adsorbent.

### Adsorption Isotherm Models

**Figure 9:**
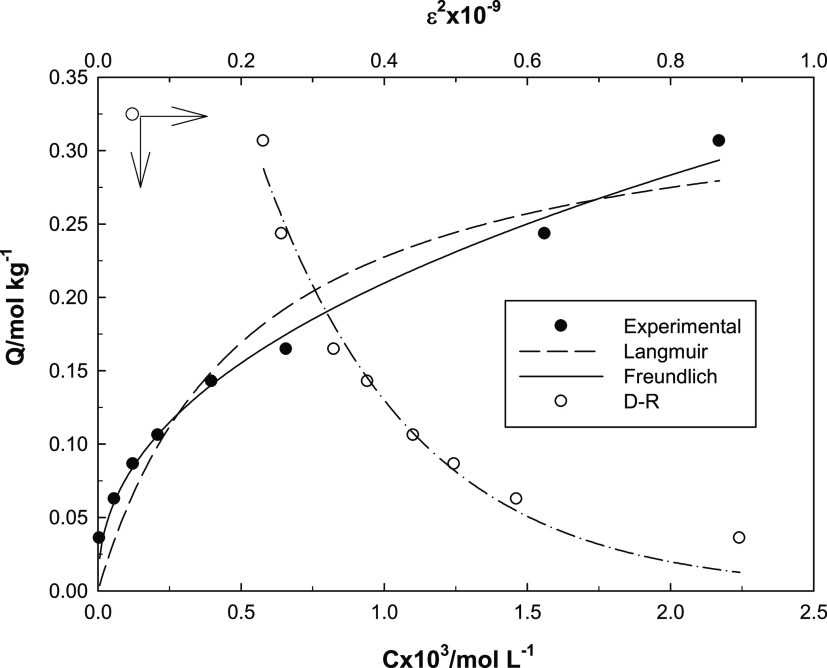
Agreement between various adsorption models and our experimentally obtained isotherm.

In adsorption studies, the analyzing of the amount of the adsorption in different concentrations is quite important. In particular, the different distributions in the concentrations of the pollutants are important for understanding the behaviors of the designed adsorbent under these conditions. Important parameters reflecting the adsorptive capacity of the designed materials can be determined via mathematical isotherm models developed for this aim. For this purpose, the adsorption ability of the newly synthesized complex at different concentrations of uranyl was investigated. The agreement with developed mathematical models of our experimental results are shown in Figure 9. The parameters provided from these models are listed in Table 1.

**Table 1:** Adsorption Parameters Obtained from Langmuir, Freundlich, and Dubinin–Radushkevich Models

Isotherm	Parameter	Value	*R*^2^
Langmuir	*X*_L_, mol kg^–1^	0.347	0.931
	*K*_L_, L mol^–1^	1899	
Freundlich	*X*_F_	4.21	0.990
	β	0.434	
DR	*X*_DR_, mol kg^–1^	0.855	0.977
	*K*_DR_, ×10^9^/mol^2^ KJ^–2^	4.71	
	*E*_DR_, kJ mol^–1^	10.3	

The compatibility of the experimental results with popular adsorption models such as Langmuir, Freundlich, and Dubinin–Raduskevich (DR)^35^ was investigated by a nonlinear regression method, and the *R*^2^ values were found to be quite high. The maximum adsorption capacity determined via the Langmuir model was reported to be 0.347 mol kg^–1^. The Langmuir model considers the surface as homogeneous and adsorption as a process that takes place through active centers. Considering the experimentally found isotherm, it can be said that the adsorption increases as the initial pH increases and a plateau is reached after a certain concentration. In this equilibrium state, the adsorption centers on the surface are now filled and the maximum adsorption capacity has been found by extrapolation of this graph.^36,37^

The parameters appearing in the Freundlich equation provide remarkable information regarding to the heterogeneity of the surface. The β value is a measure of surface heterogeneity, and smaller β values indicate the strong binding of the species in solution to the solid surface. The β value of 0.434 can be considered as proof of strong binding between the new designed material and the uranyl ion.^38^

The DR model gives information about the physical or chemical nature of adsorption, with the *E*_DR_ value, which is a measure of the adsorption energy.^39^ The *E*_DR_ value found in this study was 10 kJ mol^–1^. It implies that adsorption is chemical. It can be predicted that the adsorption occurs via chelate/complex or ion exchange of uranium over the −OH groups in the tannic acid molecule in the new designed material.

### Adsorption Kinetics

Kinetic studies provide useful information about the optimum interaction time and explanation of the mechanism of the adsorption process.

Three kinetic models to explain the adsorption process of UO_2_^2+^ ion onto MOF-Fe-Ta, pseudo-first-order (PFO) (eq 1),^40^ pseudo-second-order kinetic models (PSO) (eq 2),^41,42^ and intraparticle diffusion (IPD) (eq 3),^43^ were applied, and the results are presented in Table 2.
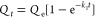
4
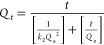
5
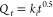
6wherein *Q*_*t*_ and *Q*_e_ represent the adsorption capacities (mol kg^–1^) at time *t* and at equilibrium, respectively. *k*_1_ (mol^–1^ kg min^–1^), *k*_2_ (mol^–1^ kg min^–1^), and *k*_i_ (mol kg^–1^min^–1^) are the PFO, PSO, and IPD rate constants, respectively.

**Table 2:** Pseudo-First-Order, Pseudo-Second-Order, and Intraparticle Diffusion Kinetic Models Parameters

Kinetic model	Parameter	Value	*R*^2^
Pseudo-first-order	*Q*_*t*_, mol kg^–1^	0.185	0.953
	*Q*_e_, mol kg^–1^	0.165	
	*k*_1_, d k^–1^	0.023	
	*H*, mol kg^–1^ min^–1^	0.038	
Pseudo-second-order	*Q*_*t*_, mol kg^–1^	0.185	0.956
	*Q*_e_, mol kg^–1^	0.186	
	*k*_2_, mol^–1^ kg min^–1^	0.150	
	*H*, mol kg^–1^ min^–1^	0.052	
Intraparticle diffusion	*k*_i_, mol kg^–1^ min^–0.5^	0.125	0.877

**Figure 10:**
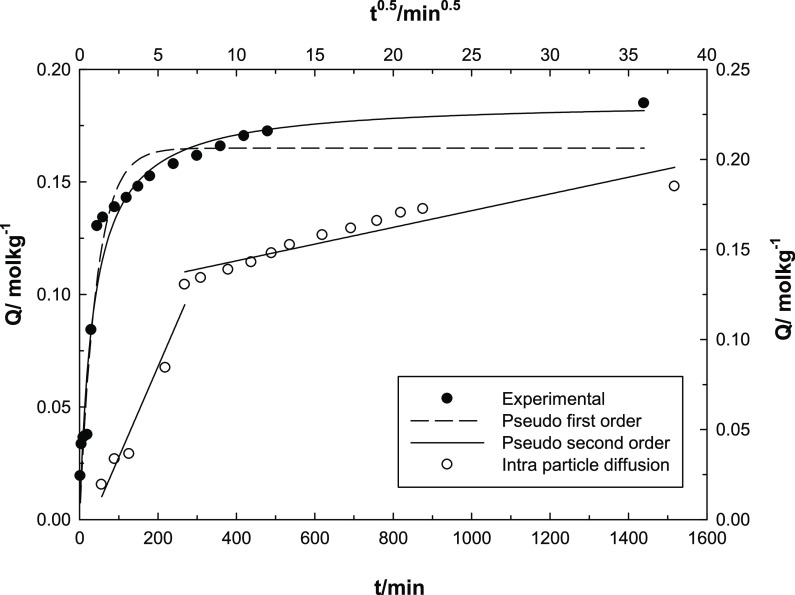
Agreement of UO_2_^2+^ adsorption kinetics with Lagergren pseudo-first-order, pseudo-second-order, and intraparticle diffusion models.

Kinetically obtained data emphasized that the adsorption of UO_2_^2+^ ions is relatively fast, and the UO_2_^2+^ ions exhibit high binding affinity to the active centers on the MOF-Fe-Ta surface. After rapid adsorption, a transitional stage occurred where there was a relatively slower adsorption rate before reaching equilibrium. In kinetic analyses, the time required to reach to the balance of the studied system was noted as 4 h (Figure 10). As a result of the comparison made between the PFO and PSO models via the correlation coefficients presented in Table 2, it can be said that our results are more compatible with PSO kinetic model. Additionally, computed *Q*_*t*_ and experimentally determined *Q*_e_ values also imply the compatibility with the PSO model. In the IPD model plot, the existence of two lines implies that adsorption occurs both on the surface and inside the surface. For that reason, as beginning, the UO_2_^2+^ ions speedly attack to the active centers on the MOF-Fe-Ta surface and then slowly and gradually penetrated the pores of the MOF-Fe-Ta.

### Adsorption Thermodynamics

**Figure 11:**
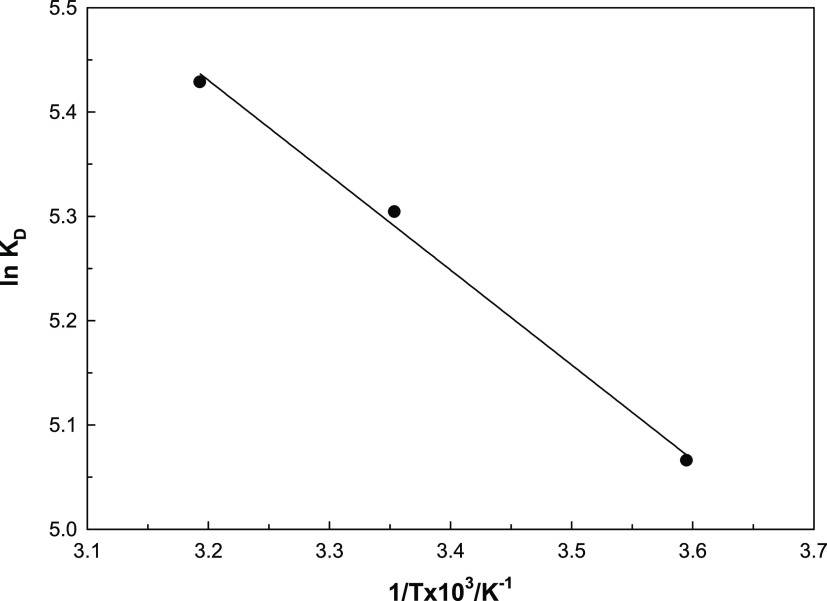
Graph showing the temperature effect on the adsorption.

For the determination and reporting of adsorption parameters, the adsorption equilibrium concentrations were measured by using different temperatures at constant concentration and other conditions, and adsorption parameters, Δ*S*, Δ*H*, and Δ*G* values were calculated by using the Van’t Hoff equation. A graphic of results is presented in Figure 11. The Δ*H*^0^ and Δ*S*^0^ values were reported as 7.56 kJ mol^–1^ and 69.3 J mol^–1^ K^–1^, respectively. The free energy values at 5, 25, and 40 °C temperatures were reported as +11.7, −13.1, and −14.2 kJ mol^–1^, respectively.

The adsorption process is a very complex phenomenon. Along with the realization of adsorption, some secondary events occur. These are events such as dehydration, ion exchange, hydrolysis on the surface, and association of water molecules.^44^ Therefore, the found adsorption thermodynamic parameters are not only the transfer of ions from the aqueous phase to the solid surface but also the parameters of the whole process. Enthalpy is generally endothermic as seen in adsorption events, while the free enthalpy value is measured negatively as an indicator of the spontaneous nature of the adsorption. A positive entropy indicates an increase in total entropy, that is, an increase in disorder. Although it is expected that the adsorption entropy will be negative due to the more ordered phase, that is, the accumulation on the solid, an increase in entropy has been observed with the effect of secondary events occurring in the total process.

### Details of the Calculations

We did restricted orbitals calculations with the B3LYP functional coupled with the all-electron SARC-DKH2 basis set for U atom and the 6-31G* basic set for other atoms. Basis functions were taken from a public repository.^45^ GAMESS-US^46^ and wxMacMolPlt 7.7^47^ software were used for calculations and visualization, respectively. Dispersion corrections D3^48^ were performed to see noncovalent interaction. Conceptual density functional theory has many applications in the various fields. This theory introduced by Parr and his team presents the following formulas to calculate the popular chemical reactivity parameters.^49,50^
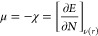
7
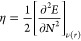
8
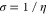
9Here μ, χ, η, and σ are chemical potential, electronegativity (absolute), hardness (absolute), and softness, respectively. *E* and *N* among the parameters appearing in the equations represent total electronic energy and total number of the electrons of the chemical system, respectively. The equations given in the following show the relation with ionization energy and electron affinities calculated in the ground state of chemical matters of the reactivity parameters.
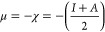
10
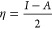
11
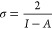
12Parr’s electrophilicity index (ω)^51^ is calculated based on absolute hardness and absolute electronegativity of chemical systems via the following equation:
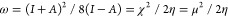
13The electroaccepting power (ω^+^) and the electrodonating power (ω^–^) of chemical systems can be predicted via the equations derived by Gazquez and co-workers.^26^ The equations derived to compute these parameters are given as follows
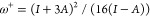
14
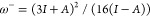
15Gomez and co-workers^27^ noticed that back-donation energy (Δ*E*_back-donation_) is dependent on the chemical hardness of molecules. The authors proposed the following equation to calculate the back-donation energy:
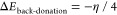
16For the estimating of the ground state ionization energy and electron affinity of the studied chemical systems, Koopmans Theorem^52^ showing that *I* = −*E*_HOMO_ and *A* = −*E*_LUMO_ for a molecule can be preferred. We also used this theorem in the prediction of *I* and *A*.

## Results and Discussion

**Figure 12:**
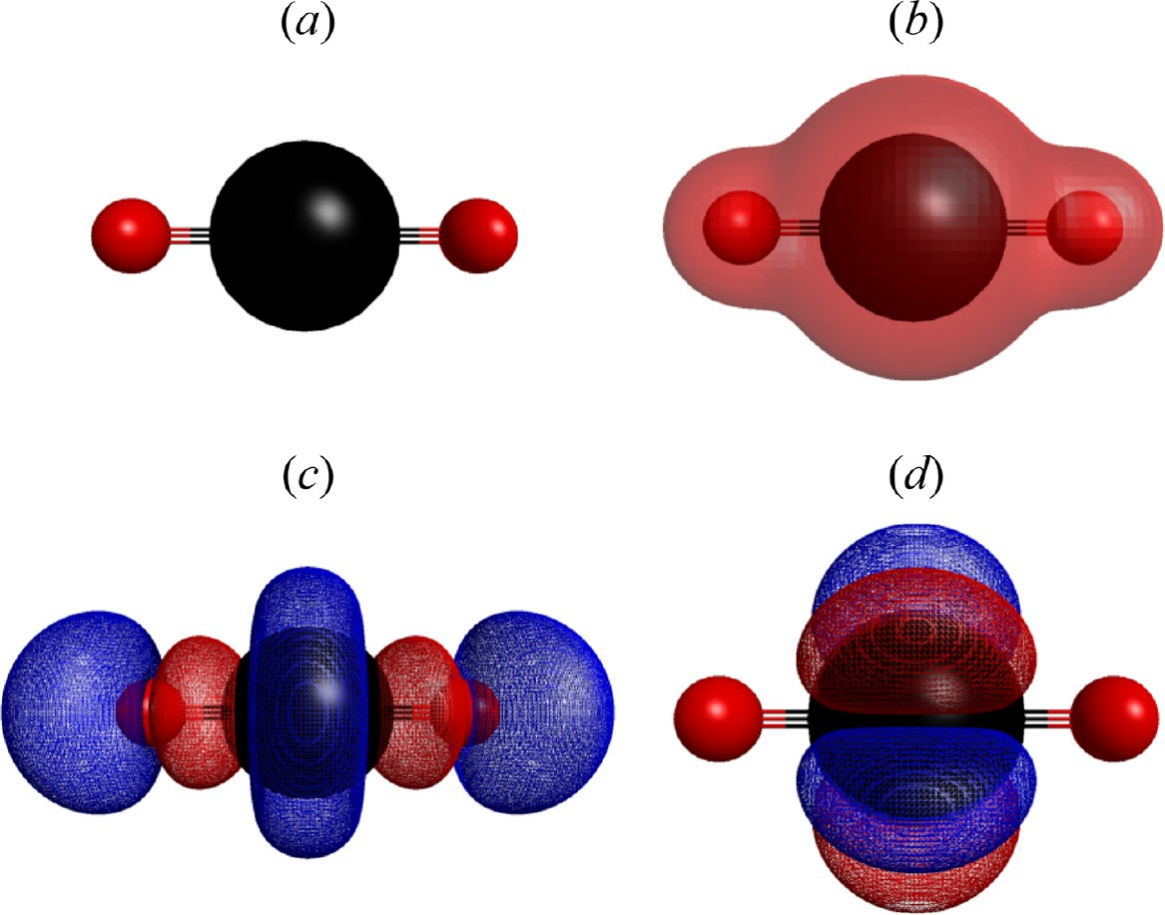
Optimized geometry (a), molecular electrostatic potential (b), and frontier orbitals HOMO (c) and LUMO (d) of the uranyl ion UO_2_^2+^.

**Figure 13:**
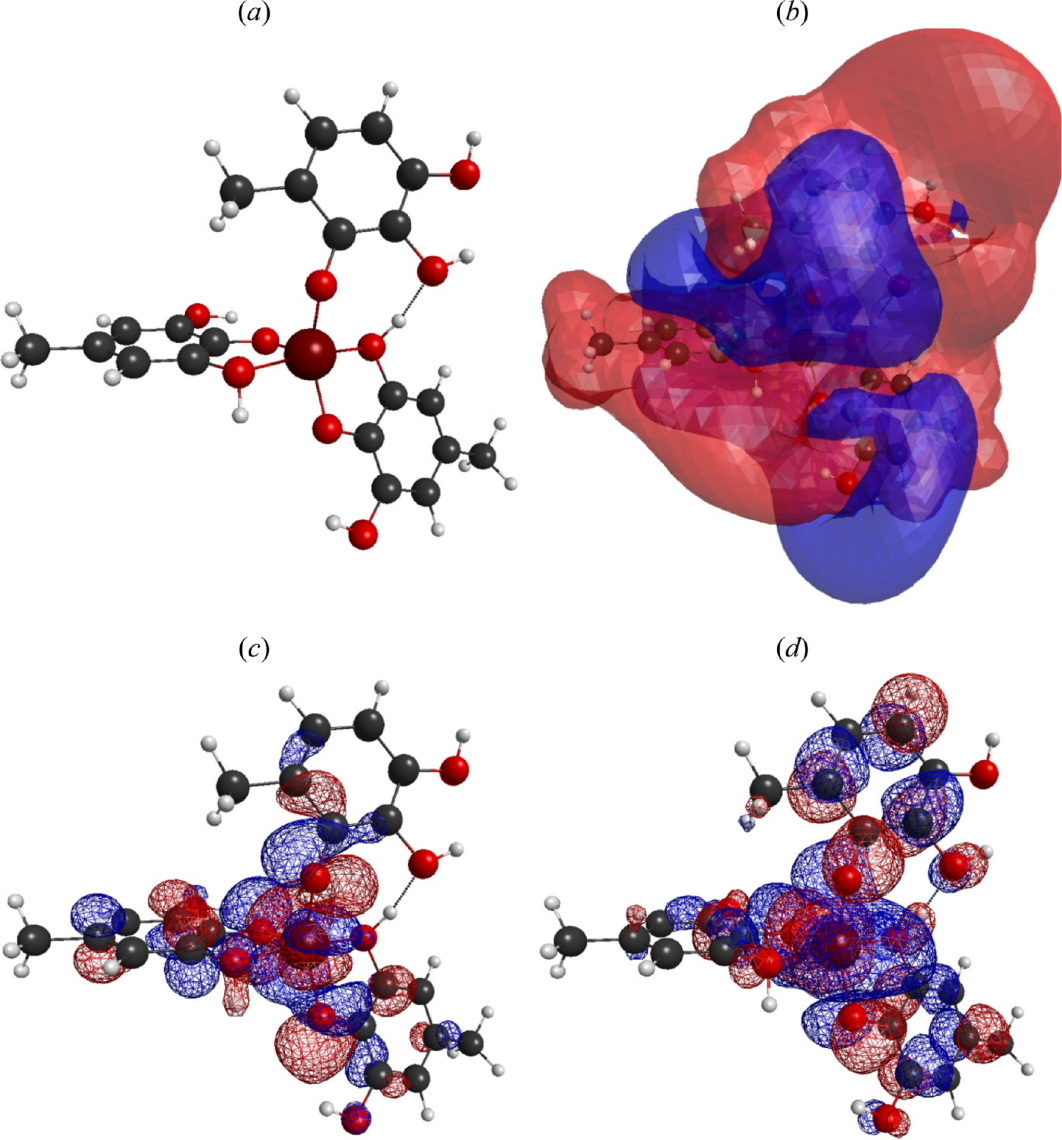
Optimized geometry (a), molecular electrostatic potential (b), and frontier orbitals HOMO (c) and LUMO (d) of the adsorbent Fe[C_7_H_7_O_3_]_3_.

**Figure 14:**
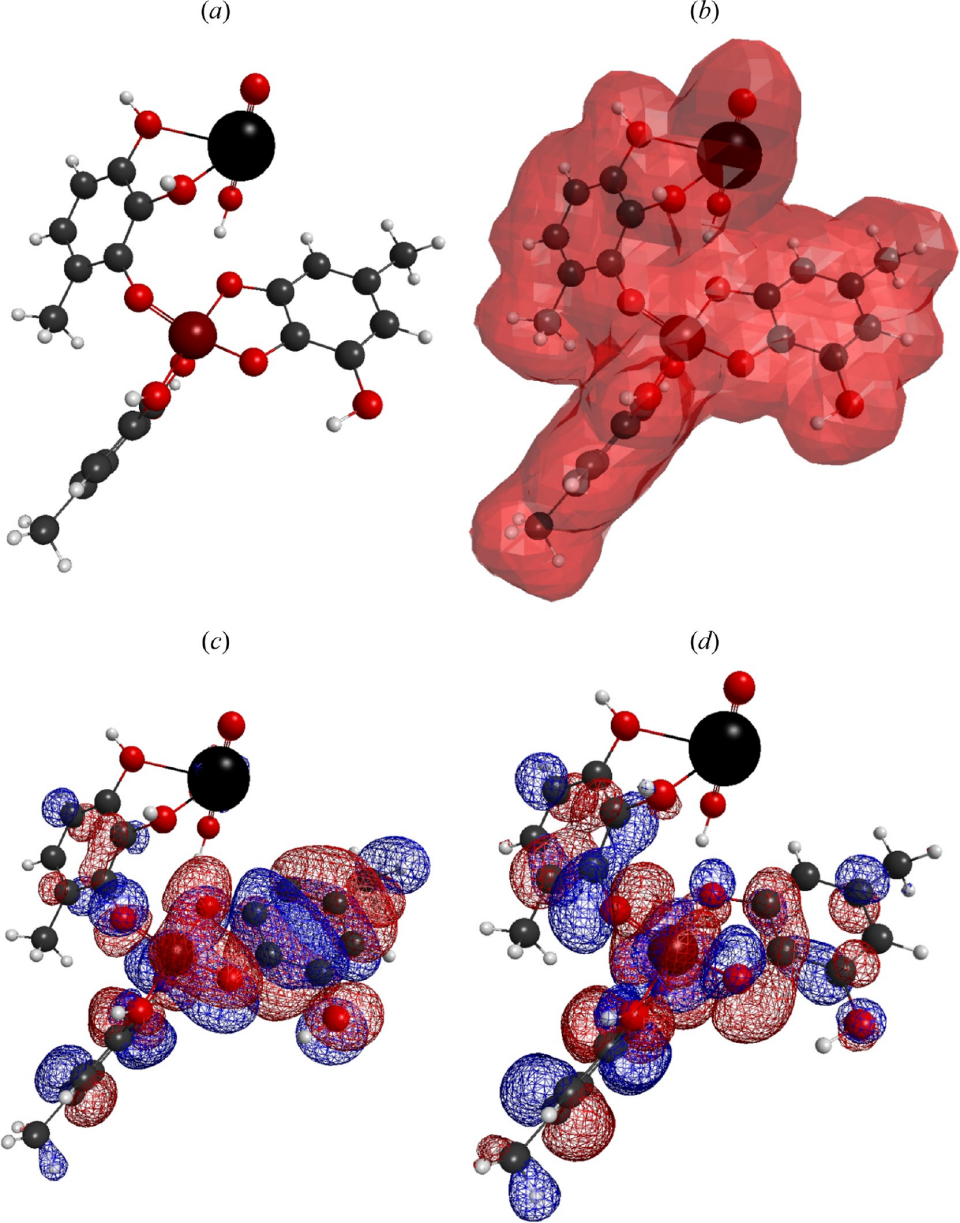
Optimized geometry (a), molecular electrostatic potential (b), and frontier orbitals HOMO (c) and LUMO (d) of the UO_2_^2+^ adsorbed on Fe[C_7_H_7_O_3_]_3_.

DFT calculations are widely preferred to see which interactions between chemical systems are more dominant and to propose the interaction mechanisms. CDFT is the branch related to chemical reactivity of DFT. In Table 3, calculated quantum chemical parameters for the adsorbent, UO_2_^2+^ ion, and complex system forming with their interaction are presented. Figure 12 presents the optimized geometry, molecular electrostatic potential, and frontier orbitals highest occupied molecular orbital (HOMO) and lowest occupied molecular orbital (LUMO) of the uranyl ion UO_2_^2+^. Figure 13 presents the HOMO, LUMO images and optimized structure of Fe[C_7_H_7_O_3_]_3_. Figure 14 shows the optimized geometry, molecular electrostatic potential, frontier orbital electrostatic potential, and frontier orbitals HOMO and LUMO of the UO_2_^2+^ adsorbed on Fe[C_7_H_7_O_3_]_3_. Chemical hardness^53,54^ represents the resistance to polarization of compounds. According to HSAB Principle,^55^ hard chemical systems are not polarizable while soft ones exhibit high polarization. The Hard and Soft Acid–Base Principle has many applications in chemistry. Toxic effects and toxicity of chemical species can be explained in light of chemical species. Some researchers have used this principle in the design of new drugs. Effective corrosion inhibitors are soft compounds. In Hard and Soft classification of Pearson, UO_2_^2+^ acts as a hard acid. In uranyl ions, the oxidation state of uranium is +6. Thanks to this charge, the uranyl ion acts as a hard acid. The interaction between the uranyl ion and Fe[C_7_H_7_O_3_]_3_ is clearly presented in Figure 14. It can be easily understood from the mentioned figure that the uranyl ion interacts with OH groups of the Fe[C_7_H_7_O_3_]_3_ structure. In the hard and soft classification of Pearson, −OH groups act as a hard base. For that reason, the interaction regarding to adsorption process is a hard–hard interaction.

**Table 3:** Calculated Characteristics of the Adsorbent, UO_2_^2+^ Ion, and Their Complex^a^

	*q*	*E*_b_, eV	HOMO, eV	LUMO, eV	η, eV	χ, eV	ω, eV	ω^–^, eV	ω^+^, eV	Δ*E*_b-d_	*D*, Debye
adsorbent	0		–4.17	–2.40	1.77	3.28	3.04	7.84	4.56	–0.44	3.78
UO_2_^2+^ ion	+2		–25.23	–21.13	4.10	23.1	65.5	142.89	119.71	–1.02	0.00
complex	+2	12.07	–11.63	–10.11	1.52	10.8	38.8	24.32	72.39	–0.38	10.20

a Adsorption energy *E*_b_ was calculated as *E*_b_ = *E*(adsorbent) + *E*(UO_2_^2+^ ion) – *E*(complex).

It can be seen from the related table that the chemical hardness value of the uranyl ion is higher than those of other studied chemical systems. The big difference between chemical hardness and electronegativity values of adsorbent and adsorbate implies a high amount of electron transfer between the adsorbent and the adsorbate. This can be predicted in light of Hardness Equalization Principle and Electronegativity Equalization Principle.^56^ The Maximum Hardness Principle^57^ states that a hard chemical system is more stable compared to soft ones. Some researchers have noted that the dipole moment can be considered as a measure of the polarizability of any chemical system. The Minimum Polarizability Principle states that chemical stability increases if polarizability is minimized. With a similar logic, Chamorro, Chattaraj, and Fuentealba^58^ proposed the minimization of electrophilicity in stable states. In a recent paper, Szentpaly and Kaya^59^ noted that the Minimum Electrophilicity Principle cannot serve as a basis for theory. The results obtained showed that the Minimum Electrophilicity Principle does not work well in this study. Binding energy (*E*_b_) calculated regarding to the interaction between the uranyl ion and Fe[C_7_H_7_O_3_]_3_ reflects the power of the interaction and the performance of the adsorbent. The binding energy value of 12.07 eV is proof of a quite powerful interaction between the uranyl ion and Fe[C_7_H_7_O_3_]_3_. This value implies that adsorption is chemical, not physical. This observation is in good agreement with the experimental data.

Performance parameters of the adsorption process are adsorption capacity, adsorption kinetic parameters, adsorption, and thermodynamic parameters of adsorption. Theoretically, binding energy and chemical hardness values calculated can give information of the efficiencies of the adsorbent. Both experimental and theoretical parameters show that the new adsorbent material is quite useful and preferable for the adsorption of UO_2_^2+^.

## Conclusion

In the present study, a novel high-performance material for the effective removal of uranyl ions from solution was synthesized and characterized. Normally, tannic acid cannot be used as an adsorbent because it is a water-soluble chemical system. However, it was seen that the Fe complex of tannic acid is quite effective in the removal of uranyl ions. The experiments showed that the new designed material has a high adsorption capacity due to the large number of hydroxide ions in its structure. The parameters affecting the adsorption process were analyzed as detailed. It was shown that adsorption occurs at the natural pH of uranium. The maximum adsorption capacity of the adsorbent was found to be quite high. The kinetics of adsorption was explained by the PSO model. The adsorption rate is quite high. The enthalpy of adsorption is endothermic, the process is with increasing entropy and the free enthalpy value is negative, that is, it is evaluated as a spontaneous process. Studies on MOFs in the literature are listed in Table 4. The interaction mechanism regarding to adsorption process was explained by DFT calculations. The stability of the studied chemical systems was predicted through popular electronic structure principles. Binding energy from the interaction between the new designed material and the uranyl ion was found as 12.07 eV. The results of theoretical and computational approaches support the experimental observations.

**Table 4:** *Q*_max_ Values for UO_2_^2+^ Adsorption to Various MOF Structures

Adsorbent type	pH	Temperature (°C)	*Q*_max_ (mol kg^–1^)	References
[Mn_5_(izdc)_3_(ox)_3_]			1.916	(60)
[UiO-66-(COOH)4-180]	4.0	20	0.528	(61)
[MIL-101-OA ]			1.189	(62)
[UiO-68]	2.5	23	0.803	(63)
[UiO-66-AO]	8.23		0.00993	(64)
MOF [MIL-101(Cr)-NH_2_]	5.5	25	0.378	(65)
[NU6CN]	5.0	25	0.821	(66)
[Zn(ADC)(4,4′-BPE)_0.5_]	6.0		1.312	(67)
MOF-76	3.0	25	1.252	(68)
[UIO-66-NH_2_]	5.5		0.482	(69)
[ZS-2/ZS-3]	5.5		0.244	(70)
MOF-Fe-Ta	4.5	25	0.347	This study

## References

[ref1] Şimşek S.; Kaya S.; Şenol Z. M.; Ulusoy H. İ.; Katin K. P.; Özer A.; Brahmia A. Theoretical and experimental insights about the adsorption of uranyl ion on a new designed Vermiculite-Polymer composite. J. Mol. Liq. 2022, 352, 118727 10.1016/j.molliq.2022.118727.

[ref2] Li Y. H.; Wang C. C.; Zeng X.; Sun X. Z.; Zhao C.; Fu H.; Wang P. Seignette salt induced defects in Zr-MOFs for boosted Pb (II) adsorption: universal strategy and mechanism insight. Chem. Eng. J. 2022, 442, 136276 10.1016/j.cej.2022.136276.

[ref3] Zhu B.; Li L.; Dai Z.; Tang S.; Zhen D.; Sun L.; Tang Z. Synthesis of amidoximated polyacrylonitrile/sodium alginate composite hydrogel beed and its use in selective and recyclable removal of U (VI). J. Radioanal. Nucl. Chem. 2022, 1–14. 10.1007/s10967-022-08233-0.35818625

[ref4] Saleh T. A. Mercury sorption by silica/carbon nanotubes and silica/activated carbon: a comparison study. J. Water Supply Res. Technol. 2015, 64 (8), 892–903. 10.2166/aqua.2015.050.

[ref5] Assaf M.; Martin-Gassin G.; Prelot B.; Gassin P. M. Driving Forces of Cationic Dye Adsorption, Confinement, and Long-Range Correlation in Zeolitic Materials. Langmuir. 2022, 38 (3), 1296–1303. 10.1021/acs.langmuir.1c03280. 35026117

[ref6] Novikau R.; Lujaniene G. Adsorption behaviour of pollutants: Heavy metals, radionuclides, organic pollutants, on clays and their minerals (raw, modified and treated): A review. J. Environ. Manage. 2022, 309, 114685 10.1016/j.jenvman.2022.114685. 35151139

[ref7] Bin-Dahman O. A.; Saleh T. A. Synthesis of polyamide grafted on biosupport as polymeric adsorbents for the removal of dye and metal ions. Biomass Conversion Biorefinery 2022, 1–14. 10.1007/s13399-022-02382-8.

[ref8] Saleh T. A.; Mustaqeem M.; Khaled M. Water treatment technologies in removing heavy metal ions from wastewater: A review. Environmental Nanotechnology, Monitoring & Management 2022, 17, 100617 10.1016/j.enmm.2021.100617.

[ref9] Vaitsis C.; Sourkouni G.; Argirusis C. Metal Organic Frameworks (MOFs) and ultrasound: A review. Ultrasonics Sonochem. 2019, 52, 106–119. 10.1016/j.ultsonch.2018.11.004. 30477790

[ref10] Kirchon A.; Feng L.; Drake H. F.; Joseph E. A.; Zhou H. C. From fundamentals to applications: a toolbox for robust and multifunctional MOF materials. Chem. Soc. Rev. 2018, 47 (23), 8611–8638. 10.1039/C8CS00688A. 30234863

[ref11] Freund R.; Zaremba O.; Arnauts G.; Ameloot R.; Skorupskii G.; Dincă M.; Bavykina A.; Gascon J.; Ejsmont A.; Goscianska J.; Kalmutzki M.; Lächelt U.; Ploetz E.; Diercks C. S.; Wuttke S. The Current Status of MOF and COF Applications. Angew. Chem., Int. Ed. 2021, 60 (45), 23975–24001. 10.1002/anie.202106259. 33989445

[ref12] Vonika K. M. Recent Advances in the Use of Metal-Organic Frameworks for Dye Adsorption. Front. Chem. 2020, 8, 1–7. 10.3389/fchem.2020.00708. 33005601 PMC7484376

[ref13] Arstad B.; Fjellvåg H.; Kongshaug K. O.; Swang O.; Blom R. Amine functionalised metal organic frameworks (MOFs) as adsorbents for carbon dioxide. Adsorption 2008, 14 (6), 755–762. 10.1007/s10450-008-9137-6.

[ref14] Tchinsa A.; Hossain M. F.; Wang T.; Zhou Y. (2021). Removal of organic pollutants from aqueous solution using metal organic frameworks (MOFs)-based adsorbents: A review. Chemosphere 2021, 284 (130), 131393 10.1016/j.chemosphere.2021.131393. 34323783

[ref15] Gülçin I.; Huyut Z.; Elmastaş M.; Aboul-Enein H. Y. Radical scavenging and antioxidant activity of tannic acid. Arab. J. Chem. 2010, 3 (1), 43–53. 10.1016/j.arabjc.2009.12.008.

[ref16] Li Y. M.; Miao X.; Wei Z. G.; Cui J.; Li S. Y.; Han R. M.; Zhang Y.; Wei W. Iron-tannic acid nanocomplexes: Facile synthesis and application for removal of methylene blue from aqueous solution. Dig. J. Nanomater. 2016, 11 (4), 1045–1061.

[ref17] Elmorsi T. M. Synthesis of Nano-Titanium Tannate as an Adsorbent for Crystal Violet Dye, Kinetic and Equilibrium Isotherm Studies. J. Environ. Prot. Sci. 2015, 6 (12), 1454–1471. 10.4236/jep.2015.612126.

[ref18] Zhang R.; Li L.; Liu J. Synthesis and characterization of ferric tannate as a novel porous adsorptive-catalyst for nitrogen removal from wastewater. RSC Adv. 2015, 5 (51), 40785–40791. 10.1039/C5RA02035B.

[ref19] Xu G.; Liu C.; Hu A.; Wang S.; Wang H. A novel synthesis of zirconium tannate with high stability: new insight into the structure of the catalyst for hydrogenation. Appl. Catal. A: Gen. 2020, 602, 117666 10.1016/j.apcata.2020.117666.

[ref20] Şimşek S. Adsorption properties of lignin containing bentonite-polyacrylamide composite for UO_2_^2+^ ions. Desalin. Water Treat. 2016, 57 (50), 23790–23799. 10.1080/19443994.2015.1135478.

[ref21] Erdem P.; Bursalı E. A.; Yurdakoç M. Preparation and Characterization of Tannic AcidResin: Study of Boron Adsorption. Environ. Prog. Sustain. Energy 2013, 32 (4), 1036 10.1002/ep.11703.

[ref22] Saleh T. A. The influence of treatment temperature on the acidity of MWCNT oxidized by HNO3 or a mixture of HNO3/H2SO4. Appl. Surf. Sci. 2011, 257 (17), 7746–7751. 10.1016/j.apsusc.2011.04.020.

[ref23] Sahiner N.; Sengel S. B.; Yildiz M. (2017). A facile preparation of donut-like supramolecular tannic acid- Fe(III) composite as biomaterials with magnetic, conductive, and antioxidant properties. J. Coord. Chem. 2017, 70 (21), 3619–3632. 10.1080/00958972.2017.1398823.

[ref24] Bulut E.; Özacar M. (2009). Rapid, facile synthesis of silver nanostructure using hydrolyzable tannin. Ind. Eng. Chem. Res. 2009, 48 (12), 5686–5690. 10.1021/ie801779f.

[ref25] Yi X.; Xu Z.; Liu Y.; Guo X.; Ou M.; Xu X. Highly efficient removal of uranium(VI) from wastewater by polyacrylic acid hydrogels. RSC Adv. 2017, 7 (11), 6278–6287. 10.1039/C6RA26846C.

[ref26] Ma D.; Wei J.; Zhao Y.; Chen Y.; Tang S. The removal of uranium using novel temperature sensitive urea-formaldehyde resin: adsorption and fast regeneration. Sci. Total Environ. 2020, 735, 139399 10.1016/j.scitotenv.2020.139399. 32492565

[ref27] Saleh T. A. Carbon nanotube-incorporated alumina as a support for MoNi catalysts for the efficient hydrodesulfurization of thiophenes. Chemical Engineering Journal 2021, 404, 126987 10.1016/j.cej.2020.126987.

[ref28] Broda E.; Gładysz-Płaska A.; Skwarek E.; Payentko V. V. Structural properties and adsorption of uranyl ions on the nanocomposite hydroxyapatite/white clay. Appl. Nanosci. 2022, 12, 1101–1111. 10.1007/s13204-021-01790-y.

[ref29] Abbott E. C.; O’Connor H. E.; Nizinski C. A.; Gibb L. D.; Allen E. W.; McDonald L. W. Thermodynamic Evaluation of the Uranyl Peroxide Synthetic Route on Morphology. J. Nucl. Mater. 2022, 561, 153533 10.1016/j.jnucmat.2022.153533.

[ref30] Lu K. T.; Zhang Y.; Wei T.; Ablott T. A.; Nguyen T. H.; Zheng R. Synthesis and characterization of a uranium oxide hydrate framework with Sr (ii) ions: structural insights and mixed uranium valences. New J. Chem. 2022, 46 (3), 1371–1380. 10.1039/D1NJ05101F.

[ref31] Roach J. M.; Manukyan K. V.; Majumdar A.; Dede S.; Oliver A. G.; Burns P. C.; Aprahamian A. Hyperstoichiometric Uranium Dioxides: Rapid Synthesis and Irradiation-Induced Structural Changes. Inorg. Chem. 2021, 60 (24), 18938–18949. 10.1021/acs.inorgchem.1c02736. 34889599

[ref32] Chen X.; Mei Q.; Yu L.; Ge H.; Yue J.; Zhang K.; Hayat T.; Alsaedi A.; Wang S. Rapid and on-site detection of uranyl ions via ratiometric fluorescence signals based on a smartphone platform. ACS Appl. Mater. Interfaces 2018, 10 (49), 42225–42232. 10.1021/acsami.8b13765. 30403334

[ref33] Arumugam K.; Burton N. A. Disproportionation of the Uranyl (V) Coordination Complexes in Aqueous Solution through Outer-Sphere Electron Transfer. Inorg. Chem. 2021, 60 (24), 18832–18842. 10.1021/acs.inorgchem.1c02575. 34847326

[ref34] Ulusoy H. İ.; Şimşek S. Removal of uranyl ions in aquatic mediums by using a new material: gallocyanine grafted hydrogel. J. Hazard. Mater. 2013, 254, 397–405. 10.1016/j.jhazmat.2013.04.004. 23669652

[ref35] Foo K. Y.; Hameed B. H. Insights into the modeling of adsorption isotherm systems. Chem. Eng. J. 2010, 156 (1), 2–10. 10.1016/j.cej.2009.09.013.

[ref36] Van Assche T. R.; Baron G. V.; Denayer J. F. An explicit multicomponent adsorption isotherm model: accounting for the size-effect for components with Langmuir adsorption behavior. Adsorption 2018, 24 (6), 517–530. 10.1007/s10450-018-9962-1.

[ref37] Saleh T. A. Isotherm, kinetic, and thermodynamic studies on Hg (II) adsorption from aqueous solution by silica-multiwall carbon nanotubes. Environmental Science and Pollution Research 2015, 22 (21), 16721–16731. 10.1007/s11356-015-4866-z. 26087931

[ref38] Nanta P.; Kasemwong K.; Skolpap W. Isotherm and kinetic modeling on superparamagnetic nanoparticles adsorption of polysaccharide. J. Environ. Chem. Eng. 2018, 6 (1), 794–802. 10.1016/j.jece.2017.12.063.

[ref39] Hu Q.; Zhang Z. Application of Dubinin–Radushkevich isotherm model at the solid/solution interface: A theoretical analysis. J. Mol. Liq. 2019, 277, 646–648. 10.1016/j.molliq.2019.01.005.

[ref40] Simonin J. P. On the comparison of pseudo-first order and pseudo-second order rate laws in the modeling of adsorption kinetics. Chem. Eng. J. 2016, 300, 254–263. 10.1016/j.cej.2016.04.079.

[ref41] Islam M. A.; Chowdhury M. A.; Mozumder M. S. I.; Uddin M. T. Langmuir Adsorption Kinetics in Liquid Media: Interface Reaction Model. ACS omega 2021, 6 (22), 14481–14492. 10.1021/acsomega.1c01449. 34124471 PMC8190925

[ref42] Saleh T. A.; Elsharif A. M.; Bin-Dahman O. A. Synthesis of amine functionalization carbon nanotube-low symmetry porphyrin derivatives conjugates toward dye and metal ions removal. J. Mol. Liq. 2021, 340, 117024 10.1016/j.molliq.2021.117024.

[ref43] Valderrama C.; Gamisans X.; De las Heras X.; Farran A.; Cortina J. L. Sorption kinetics of polycyclic aromatic hydrocarbons removal using granular activated carbon: intraparticle diffusion coefficients. J. Hazard. Mater. 2008, 157 (2–3), 386–396. 10.1016/j.jhazmat.2007.12.119. 18308468

[ref44] Şimşek S.; Şenol Z. M.; Ulusoy H. I. Synthesis and characterization of a composite polymeric material including chelating agent for adsorption of uranyl ions. J. Hazard. Mater. 2017, 338, 437–446. 10.1016/j.jhazmat.2017.05.059. 28595158

[ref45] Pritchard B. P.; Altarawy D.; Didier B.; Gibson T. D.; Windus T. L. A New Basis Set Exchange: An Open, Up-to-date Resource for the Molecular Sciences Community. J. Chem. Inf Model. 2019, 59 (11), 4814–4820. 10.1021/acs.jcim.9b00725. 31600445

[ref46] Barca G. M. J.; Bertoni C.; Carrington L.; Datta D.; De Silva N.; Deustua J. E.; Fedorov D. G.; Gour J. R.; Gunina A. O.; Guidez E.; Harville T.; Irle S.; Ivanic J.; Kowalski K.; Leang S. S.; Li H.; Li W.; Lutz J. J.; Magoulas I.; Mato J.; Mironov V.; Nakata H.; Pham B. Q.; Piecuch P.; Poole D.; Pruitt S. R.; Rendell A. P.; Roskop L. B.; Ruedenberg K.; Sattasathuchana T.; Schmidt M. W.; Shen J.; Slipchenko L.; Sosonkina M.; Sundriyal V.; Tiwari A.; Galvez Vallejo J. L.; Westheimer B.; Włoch M.; Xu P.; Zahariev F.; Gordon M. S. Recent developments in the general atomic and molecular electronic structure system. J. Chem. Phys. 2020, 152, 154102 10.1063/5.0005188. 32321259

[ref47] Bode B. M.; Gordon M. S. Macmolplt: A graphical user interface for GAMESS. Journal of Molecular Graphics and Modelling 1998, 16, 133–138. 10.1016/S1093-3263(99)00002-9. 10434252

[ref48] Grimme S.; Antony J.; Ehrlich S.; Krieg H. A consistent and accurate ab initio parametrization of density functional dispersion correction (DFT-D) for the 94 elements H-Pu. J. Chem. Phys. 2010, 132, 154104 10.1063/1.3382344. 20423165

[ref49] Conceptual density functional theory and its application in the chemical domain; Islam N., Kaya S.,Eds.; CRC Press, 2018.

[ref50] Kaya S.; Kaya C.; Guo L.; Kandemirli F.; Tüzün B.; Uğurlu İ.; Saraçoğlu M. Quantum chemical and molecular dynamics simulation studies on inhibition performances of some thiazole and thiadiazole derivatives against corrosion of iron. J. Mol. Liq. 2016, 219, 497–504. 10.1016/j.molliq.2016.03.042.

[ref51] Parr R. G.; Szentpály L. V.; Liu S. Electrophilicity index. J. Am. Chem. Soc. 1999, 121 (9), 1922–1924. 10.1021/ja983494x.

[ref52] Koopmans T. Über die Zuordnung von Wellenfunktionen und Eigenwerten zu den einzelnen Elektronen eines Atoms. physica 1934, 1 (1–6), 104–113. 10.1016/S0031-8914(34)90011-2.

[ref53] Kaya S.; Kaya C. A new equation for calculation of chemical hardness of groups and molecules. Mol. Phys. 2015, 113 (11), 1311–1319. 10.1080/00268976.2014.991771.

[ref54] Kaya S.; Kaya C. A new method for calculation of molecular hardness: a theoretical study. Comput. Theor. Chem. 2015, 1060, 66–70. 10.1016/j.comptc.2015.03.004.

[ref55] Chattaraj P. K.; Lee H.; Parr R. G. HSAB principle. J. Am. Chem. Soc. 1991, 113 (5), 1855–1856. 10.1021/ja00005a073.

[ref56] Kaya S.; Kaya C. A new equation based on ionization energies and electron affinities of atoms for calculating of group electronegativity. Comput. Theor. Chem. 2015, 1052, 42–46. 10.1016/j.comptc.2014.11.017.

[ref57] Pearson R. G. (1993). The principle of maximum hardness. Acc. Chem. Res. 1993, 26 (5), 250–255. 10.1021/ar00029a004.

[ref58] Chamorro E.; Chattaraj P. K.; Fuentealba P. (2003). Variation of the electrophilicity index along the reaction path. J. Phys. Chem. A 2003, 107 (36), 7068–7072. 10.1021/jp035435y. 26313132

[ref59] von Szentpály L.; Kaya S.; Karakuş N. Why and when is electrophilicity minimized? New theorems and guiding rules. J. Phys. Chem. A 2020, 124 (51), 10897–10908. 10.1021/acs.jpca.0c08196. 33301330

[ref60] Zhang T.; Ling B. K.; Hu Y. Q.; Han T.; Zheng Y. Z. An anionic manganese (ii) metal-organic framework for uranyl adsorption. CrystEngComm 2019, 21 (26), 3901–3905. 10.1039/C9CE00603F.

[ref61] Wang X.; Chen L.; Bai Z.; Zhang D.; Guan J.; Zhang Y.; Shi C.; Diwu J. In vivo uranium sequestration using a nanoscale metal–organic framework. Angew. Chem. Int. 2021, 133 (3), 1670–1674. 10.1002/ange.202012512. 33029917

[ref62] Wu H.; Chi F.; Zhang S.; Wen J.; Xiong J.; Hu S. Control of pore chemistry in metal-organic frameworks for selective uranium extraction from seawater. Micropor. Mesopor. Mater. 2019, 288, 109567 10.1016/j.micromeso.2019.109567.

[ref63] Carboni M.; Abney C. W.; Liu S.; Lin W. Highly porous and stable metal–organic frameworks for uranium extraction. Chem. Sci. 2013, 4 (6), 2396–2402. 10.1039/c3sc50230a.

[ref64] Chen L.; Bai Z.; Zhu L.; Zhang L.; Cai Y.; Li Y.; Liu W.; Wang Y.; Chen L.; Diwu J.; Wang J.; Chai Z.; Wang S. Ultrafast and efficient extraction of uranium from seawater using an amidoxime appended metal–organic framework. ACS Appl. Mater. Interfaces. 2017, 9 (38), 32446–32451. 10.1021/acsami.7b12396. 28910070

[ref65] Bai Z.-Q.; Yuan L.-Y.; Zhu L.; Liu Z.-R.; Chu S.-Q.; Zheng L.-R.; Zhang J.; Chai Z.-F.; Shi W.-Q. Introduction of amino groups into acid-resistant MOFs for enhanced U (VI) sorption. J. Mater. Chem. 2015, 3 (2), 525–534. 10.1039/C4TA04878D.

[ref66] Xie Y.; Chen C.; Ren X.; Tan X.; Song G.; Chen D.; Alsaedi A.; Hayat T. Coupling g-C3N4 nanosheets with metal-organic frameworks as 2D/3D composite for the synergetic removal of uranyl ions from aqueous solution. J. Colloid Interface Sci. 2019, 550, 117–127. 10.1016/j.jcis.2019.04.090. 31059894

[ref67] Liu S.; Luo M.; Li J.; Luo F.; Ke L.; Ma J. Adsorption equilibrium and kinetics of uranium onto porous azo-metal–organic frameworks. J. Radioanal. Nucl. Chem. 2016, 310 (1), 353–362. 10.1007/s10967-016-4852-z.

[ref68] Yang W.; Bai Z.-Q.; Shi W.-Q.; Yuan L.-Y.; Tian T.; Chai Z.-F.; Wang H.; Sun Z.-M. MOF-76: from a luminescent probe to highly efficient U VI sorption material. Chem. Commun. 2013, 49 (88), 10415–10417. 10.1039/C3CC44983A. 24079004

[ref69] Luo B. C.; Yuan L. Y.; Chai Z. F.; Shi W. Q.; Tang Q. U (VI) capture from aqueous solution by highly porous and stable MOFs: UiO-66 and its amine derivative. J. Radioanal. Nucl. Chem. 2016, 307 (1), 269–276. 10.1007/s10967-015-4108-3.

[ref70] Zheng T.; Yang Z.; Gui D.; Liu Z.; Wang X.; Dai X.; Wang S. Overcoming the crystallization and designability issues in the ultrastable zirconium phosphonate framework system. Nat. Commun. 2017, 8 (1), 1–11. 10.1038/ncomms15369. 28555656 PMC5459948

